# CCR5 as a Natural and Modulated Target for Inhibition of HIV

**DOI:** 10.3390/v6010054

**Published:** 2013-12-30

**Authors:** Bryan P. Burke, Maureen P. Boyd, Helen Impey, Louis R. Breton, Jeffrey S. Bartlett, Geoff P. Symonds, Gero Hütter

**Affiliations:** 1 Calimmune, Inc., Los Angeles, California, CA 90024, USA; E-Mails: bryan.burke@calimmuneinc.com (B.P.B.); maureen.boyd@calimmuneinc.com (M.P.B.); helen.impey@calimmuneinc.com (H.I.); louis.breton@calimmuneinc.com (L.R.B.); jeffrey.bartlett@calimmuneinc.com (J.S.B.); 2 Institute of Transfusion Medicine and Immunology, Medical Faculty Mannheim, Heidelberg University, German Red Cross Blood Service Baden-Württemberg-Hessen, 68167 Mannheim, Germany; E-Mail: gero.huetter@medma.uni-heidelberg.de; 3 Cellex GmbH, 01307 Dresden, Germany

**Keywords:** CCR5, C46, gene therapy, HIV, stem cell transplantation

## Abstract

Human immunodeficiency virus type 1 (HIV-1) infection of target cells requires CD4 and a co-receptor, predominantly the chemokine receptor CCR5. CCR5-delta32 homozygosity results in a truncated protein providing natural protection against HIV infection—this without detrimental effects to the host—and transplantation of CCR5-delta32 stem cells in a patient with HIV (“Berlin patient”) achieved viral eradication. As a more feasible approach gene-modification strategies are being developed to engineer cellular resistance to HIV using autologous cells. We have developed a dual therapeutic anti-HIV lentiviral vector (LVsh5/C46) that down-regulates CCR5 and inhibits HIV-1 fusion via cell surface expression of the gp41-derived peptide, C46. This construct, effective against multiple strains of both R5- and X4-tropic HIV-1, is being tested in Phase I/II trials by engineering HIV-resistant hematopoietic cells.

## 1. CCR5 and the CCR5-Delta32 Deletion

The entry of human immunodeficiency virus type 1 (HIV-1) into target cells requires both CD4 and a co-receptor, predominantly the chemokine receptor CCR5. A 32 base pair deletion in the CCR5 gene results in a truncated protein that is insufficient for HIV entry. CCR5-delta32 homozygosity provides natural protection against HIV infection without detrimental effects to the host, further commented on below [1].

## 2. A First Case of a “Natural” Gene Therapy

In 2009, Hütter and colleagues described successful hematopoietic stem cell transplantation (SCT) in an HIV-1 infected patient by transferring CCR5-delta32 donor derived cells that harbor a natural resistance against HIV infection. These hematopoietic stem cells engrafted, proliferated and differentiated into mature myeloid and lymphoid cells. At present the patient is more than five years post allogeneic transplantation without the requirement of any antiretroviral treatment. Analyzing peripheral blood cells and different tissue samples including gut, liver, and brain, no viral RNA load or proviral DNA could be detected [2,3].

## 3. Benefits and Risks of Transplanting CCR5 Deficient Stem Cells

Recipients of organ allografts homozygous for CCR5-delta32 show longer survival of transplant function than those with other genotypes. This has been shown for renal and liver transplants suggesting that patients with CCR5-delta32 might be candidates for a reduced immunosuppressive therapy [4,5]. Consequently, interaction and blockade of the CCR5 receptor may also reduce alloantigen-specific T lymphocyte proliferation, and may be effective in preventing acute and chronic rejection of the allograft [6]. Furthermore, the presence of the CCR5-delta32 allele represents a protective factor in terms of the risk of developing graft-*versus*-host disease (GvHD) after allogeneic SCT [7]. Taken together, the presence of the CCR5-delta32 allele in recipients of allografts constitutes an independent and protective factor associated with a decreased risk of GvHD and graft rejection. However, the mechanism of this beneficial effect of the deletion regarding GvHD is not known.

In the past there was much speculation about the association of the CCR5-delta32 genotype and other diseases with chronic autoimmune inflammation. The results of these retrospective studies are controversial. No association or beneficial effect was observed in diabetes mellitus type 1, asthma, rheumatoid arthritis, and Behcet’s disease [8,9,10,11]. There have been reports of individuals with the CCR5-delta32 homozygous genotype being associated with an increased risk of symptomatic West Nile virus infection [12].

Thus overall, there is no evidence that the CCR5-delta32 genotype is associated with significant negative co-morbidities or risks in terms of transplantation, and the presence of the CCR5-delta32 genotype should not have negative implications for the recipient of a CCR5 targeted therapy.

## 4. Challenges in Repeating the “Berlin Patient”

### 4.1. HLA Matched Related or Unrelated Donors

Over 16,000,000 people are already registered worldwide as stem cell donors. Based on a 10/10 allele HLA-match, the probability in finding a matching donor is over 80%, and there is commonly more than 1 donor and sometimes more than 100 donors available for each patient. According to the frequency of around 1% homozygous CCR5-delta32 Caucasians, there is a small but reasonable chance to find both an HLA-identical donor without CCR5 surface expression.

The major limitations are that donors are not generally tested for CCR5 genotype, and there is commonly not enough time to complete the screening process. Therefore, Hütter’s group organized a workshop in 2009 bringing together leading European stem cell registries for a discussion of the possibilities and limitations of a CCR5-based donor screening. The meeting came to the agreement to support further attempts to use CCR5-delta32 deleted stem cells in appropriate candidates. However, none of these registries initiated a program of pre-emptive CCR5 genotyping [13].

With a grant from the Bill & Melinda Gates foundation, Hütter’s group was able to carry on 18 additional donor searches requesting units for transplantation (Table 1).

**Table 1:** Summary of 18 patient/donor screening series searching for a CCR5-delta32 homozygote stem cell donor.

Start of Screening	Gender	Age	Diagnosis	Location	Registered Donors	CCR5 Results WT/HG/HC	Status
03/09	male	adult	NHL	Freiburg, Germany	>1	ND	died before Tx
03/09	female	3	DBA	Heidelberg, Germany	120	103/17/1 ^a^	stopped
07/09	male	adult	MDS	Lausanne, Switzerland	1	1/-/-	Tx with WT
11/09	male	29	NHL	Mainz, Germany	1	1/-/-	Tx with WT
12/09	male	15	AML	Jerusalem, Israel	6	ND/ND/3 ^a^	Tx with untested, died after Tx
01/10	male	50	CMML	Berlin, Germany	60	25/5/0	Tx with WT
03/11	male	59	NA	Berlin, Germany	1 ^b^	1/-/-	NA
05/11	male	adult	NHL	Mannheim, Germany	ND	ND	Tx cancelled
06/11	male	14	KS	Dublin, Ireland	ND	ND	stopped
08/11	male	42	AML	Hamburg, Germany	1	1/-/-	Tx with wt
01/11	male	48	MDS	Frankfurt, Germany	3^c^	ND	stopped
01/11	female	70	AML	Frankfurt, Germany	ND	ND	died before Tx
06/12	male	61	CMML	Frankfurt, Germany	2	ND	Tx chancelled
05/12	male	adult	AML	Münster, Germany	30	7/2/1	died before Tx
08/12	male	adult	AML	Hamburg, Germany	>10	4/4/0	lost to follow up
02/13	male	adult	AML	Cagliari, Italy	3 ^b^	3/0/0	pending
03/13	female	40	NHL	Basel, Switzerland	35	24/1/1	pending
03/13	male	51	NHL	Münster, Germany	>30	38/3/1 ^c^	pending

AML = acute myeloblastic leukemia; CMML = chronic myelomoncytic leukemia; DBA = Diamond-Blackfan anemia; HC = homozygotes; HG = heterozygotes; KS = Kaposi’s sarcoma; MDS = myelodysplastic syndrome; ND = not done; NHL = non Hodgkin’s lymphoma; Tx = transplantation; WT = wild type; ^a^ cord blood unit with 3/6 HLA-match; ^b^ matched related donor; ^c^ HLA mismatch.

Unfortunately, in none of these cases could a CCR5-negative stem cell unit be administered. In three cases the search found at least 1 unit with the CCR5-delta32 homozygous genotype; however, one patient died prior to initiation of the transplantation procedure, the decision for transplantation is pending for the second case, and the CCR5 negative unit had a HLA mismatch for the third patient.

Furthermore, Hutter’s group initiated a CCR5 genotype pre-screen program for the “German Red Cross Donor Registry” of Mannheim, Germany, which currently has tested approximately 8,000 of 40,000 adult unrelated stem cell donors. Together with the cord blood bank of Stemcyte, Covina, CA, USA (>25,000 units) and the M.D. Anderson CB Bank, Houston, TX, USA (>10,000 units), these repositories represent the largest potential source of CCR5 negative stem cells for transplantation.

### 4.2. Cord Blood: An Alternative Stem Cell Source

As an alternative to HLA matched related or unrelated donors, cord blood units are an interesting stem cell source. Since 2001 R. Chow (founder of StemCyte Inc.) and L. Petz built up a database with more than 25,000 cord blood units genotyped for the CCR5-delta32 deletion, and over 300 of these UCB units have already been identified as being CCR5 negative. This would be sufficient to provide a 4/6 HLA matched unit with a 73.6% probability to Caucasian paediatric patients, and 27.9% for Caucasian adults in the case that a minimal cell dose of ≥2.5 × 10^7^ total nucleated cell count (TNC)/kg is accepted [14].

However, since Stemcyte started the CCR5 testing in 2001, there were no CCR5-delta 32 homozygous UCB units administered up to 2011. The reason is probably due to the good access of perfectly matched stem cells from adult donors. Some countries, like Germany, do not use UCB in significant amounts. Some transplant centers are anxious that a delayed engraftment of UCB and the consequent higher risk of opportunistic infections during aplasia would narrow the success of the transplant procedure.

To overcome these limitations, strategies to increase the probability of administration of UCB are required. One option could be the so called “dual transplant” method consisting of the co-infusion of one UCB unit with a relatively low number of highly T-depleted mobilized CD34^+^ cells from an adult third party donor, after myeloablative conditioning regimen [15].

This approach has several advantages:

1. High accessibility of a haploidentical donor that bridges the time until the CCR5-negative UCB engrafts.
2. Increased probability of finding matching UCB due to the reduction of the required CD34^+^ cell dose. Lowering the required cell dose from 2.5 to 1.0 × 10^7^ TNC/kg will increase the probability from 27.9% to 82.1%.
3. Reduced time of aplasia due to the haploidentical proportion of the transplant.
4. Finally, after engraftment, the UCB unit will take over the myeloid function while the haploidentical part disappears after a period of time resulting in a 100% chimerism of CCR5 depleted and HIV resistant peripheral blood cells.

Competition between grafts of various sources is commonly observed after double UCB transplantation and a similar competition phenomenon occurs after combined haploidentical/UCB transplantation. Immunologic mechanisms likely play a role in the UCB cord blood dominance and in this regard the T-cell depletion of the haplograft may be important but probably not essential [16]. This approach has already been performed in some selected patients (publication of first results is in progress) [17,18]. Unfortunately, one of these patients died due to relapse of the malignancy 2½ months after the transplantation procedure [19]. Due to the difficulties in finding a second candidate for SCT from an adult unrelated donor, the meeting suggested the initiation of a program to promote the idea of combined haploidentical/CCR5-negative UCB based on the experiences of the University of Utrecht, the Netherlands [20].

## 5. Limitations of the CCR5-Delta32 Approach

During transmission of HIV, CCR5 is the preferred co-receptor for cell entry. However, during the time course of HIV infection, the virus is able to change its tropism to other chemokine co-receptors, such as CXCR4, and the role of CCR5 in maintaining HIV infection is still unclear. The “Berlin patient” harbored a CXCR4 tropic variant before transplantation that did not emerge after discontinuation of antiretroviral therapy, a phenomenon that cannot yet be explained [20,21].

Secondly, there is a broad discussion about sterilizing cure in the “Berlin patient”. However, current investigations and several attempts with different techniques from tissues, cerebral fluid, and blood have led to contradicting results. However, the patient is well, more than 5 years off antiretroviral medication, without HIV related symptoms, without viral replication showing a perfect clinical remission of the infection [22].

Most recently, the report of a long-term control of HIV replication in two patients receiving an allogeneic SCT with a CCR5 wild type allo-graft (“Boston Patients”) has instigated new questions. After SCT these patients showed a profound reduction of the viral reservoir, declining HIV antibody levels, and ongoing engraftment during the follow-up period of several years similar to the “Berlin Patient” [23]. Finally, in 2013 antiretroviral medication was discontinued and none of the patients developed a rebound [24]. Transplantation has led to a replacement of the original immune system, antiretroviral therapy had protected the new engrafting cells from re-infection, and it is likely that GvHD has cleared residual viral reservoirs in the host. In conclusion, this could mean that CCR5 withdrawal in an allogeneic stem cell setting is not mandatory for a successful viral eradication. However, the exact mechanism and role of antiretroviral therapy, chemotherapy, and GvHD in this outcome will need to be determined.

## 6. Modulation of CCR5 Expression

Modulation of CCR5 expression in autologous cells is actively being pursued by multiple research teams to develop alternative therapies for treatment of HIV-1 infection with the potential of long-term therapeutic benefit. Gene therapeutic approaches targeting the disruption of CCR5 expression have been developed using ribozymes, intrabodies, and RNA interference that inhibit cell surface CCR5 expression; more recently, nucleases have been shown effective at disrupting the CCR5 gene (an approach undertaken by Sangamo) [25]. In general an approach targeting viral entry is preferred. We are currently testing a strategy that would protect the immune system and potentially allow patients to come off their life-time of drugs while mitigating the risks associated with the allo-SCT used in both the “Berlin patient” and “Boston patients”. Importantly, this approach is not only amenable to HIV-1 infected patients with malignancy, but to any HIV-1 infected individual due to the use of autologous cells. In this approach, a lentiviral vector is used to introduce a gene encoding a short hairpin RNA (shRNA) against CCR5 into an individual’s autologous hematopoietic stem/progenitor cells (HSPC) to protect those cells and their progeny from HIV. This shRNA, termed sh1005 (sh5), was identified by screening a library of more than one thousand candidate shRNAs targeting CCR5 [26]. Once expressed within the cell, the shRNA is processed by cellular machinery into a small interfering RNA (siRNA) for stable inhibition of CCR5 through a mechanism known as RNA interference (RNAi). RNAi has recently emerged as a powerful therapeutic approach towards a number of human diseases. Qin *et al.*, first described *in vitro* success constructing a lentivirus-based vector to introduce sh5 into human peripheral blood T lymphocytes, and later demonstrated stable expression of sh5 in non-human primates following transplantation of *ex vivo* modified CD34^+^ HSPC [27,28]. 14 months after transplant, they were able to detect lymphocytes expressing sh5 and consistent down-regulation of the CCR5 receptor. *Ex vivo* studies showed that the gene-modified cells were less susceptible to Simian Immunodeficiency Virus (SIV) infection. Later, Liang *et al*., reported over 90% reduction in CCR5 mRNA levels and resistance to R5 tropic HIV-1 in macrophages differentiated *in vitro* from fetal liver-derived CD34^+^ HSPC transduced with a lentiviral vector encoding sh5 [29].

*In vivo* evaluation in a humanized bone marrow/liver/thymus (BLT) mouse model by Shimizu *et al*., demonstrated stable knockdown of human CCR5 by sh5 [30]. When introduced into human fetal liver-derived CD34^+^ HSPC by lentiviral vector transduction and transplanted into irradiated BLT mice, sh5 mediated CCR5 knockdown had no apparent adverse effects on T-cell development and suggested the potential for long-term hematopoietic reconstitution.

We have since continued pre-clinical development of sh5 within the context of a lentiviral vector and confirmed both efficacy and lack of toxicity.

## 7. Addition of a Second Inhibitory Element

It is well accepted that highly active antiretroviral therapy uses 2–3 drugs to effectively suppress HIV replication and mitigate the development of resistance. For this reason we have introduced a second element into its anti-HIV lentiviral vector construct. This second element is an HIV fusion inhibitor, which inhibits HIV-1 fusion via cell surface expression of a gp41-derived peptide. This peptide, known as C46, is comprised of 46 amino acids, 36 of them corresponding to the fusion-inhibitory peptide C36 (T-20/Enfuvirtide) [31]. It is expressed as a fusion protein with an N-terminal signal peptide, which targets the peptide through the endoplasmic reticulum to the cell surface and a C-terminal linker followed by a membrane-spanning domain. This moiety was originally developed by Dorothee von Laer’s group in Austria, which has generated strong pre-clinical data in tissue culture and in animal models indicating that C46 is very efficient at inhibiting HIV replication and its pathogenic effects [31,32]. In a Phase I clinical trial, 10 advanced HIV^+^ subjects received T lymphocytes transduced with a retroviral vector that encoded C46 [33]. This treatment was shown to be safe with ongoing detection of gene-marked cells in the peripheral blood, lymph nodes and bone marrow throughout a 1 year follow up period. Recently, the gene encoding C46 was also used to modify CD34^+^ HSPC in a non-human primate model and could mediate effective protection effect against Simian-Human Immunodeficiency Virus (SHIV) challenge [34].

**Figure 1:**
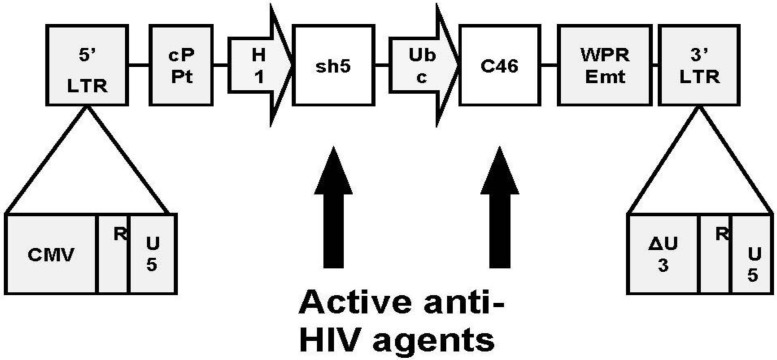
Lentiviral construct LVsh5/C46. LVsh5/C46 is a self-inactivating (SIN) lentiviral vector that lacks all viral coding sequences (*gag*, *pol*, and *env* genes deleted) which may give rise to the formation of replication-competent retrovirus or immunogenic peptides and is also devoid of all retroviral enhancer-promoter sequences that are known to be involved in insertional mutagenesis by related gammaretroviral-derived vectors. The internal promoters were chosen from human genes that show expression in hematopoietic stem/progenitor cells as well as T lymphocytes and macrophages, as required for anti-HIV therapy. Promoters were also chosen to direct appropriate levels of gene expression so as not to interfere with endogenous cellular processes. 5' LTR (long terminal repeat), derived from HIV-1 with the U3 region replaced with the cytomegalovirus (CMV) promoter/enhancer; 3' LTR, derived from HIV-1 with a 133bp deletion in the U3 region; cPPT, central polypurine tract; H1, human H1 RNA promoter; Ubc, human ubiquitin promoter; WPREmt, mutant woodchuck hepatitis virus post-transcriptional response element.

Our team has incorporated both the gene encoding the CCR5-specific shRNA (sh5) and the gene encoding C46 into a dual therapeutic lentiviral construct termed LVsh5/C46 (Figure 1).

**Figure 2:**
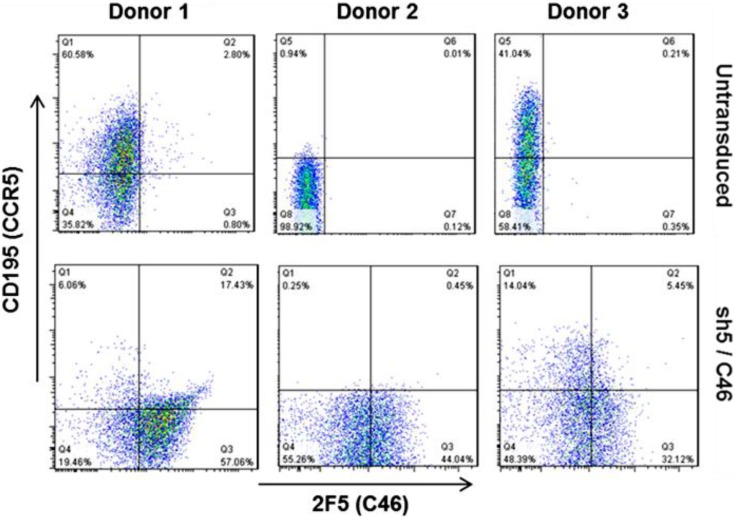
LVsh5/C46 introduction into target cells and simultaneous expression of C46 and knockdown of CCR5. Peripheral blood mononuclear cells (PBMC) were transduced with LVsh5/C46 (lower panel), or left untransduced (upper panel). C46 was detected by 2F5 monoclonal antibody, and CCR5 was detected by staining with anti-CD195 (CCR5) antibody. Cells from 3 independent donors are shown. Donor 2 was homozygous for CCR5-delta32 genotype and expresses no CCR5.

The combined approach of sh5-mediated down-regulation of CCR5 and C46-mediated inhibition of fusion, appears to be significantly more effective at engineering cellular resistance to HIV than approaches utilizing single agents. The LVsh5/C46 construct has undergone extensive pre-clinical testing for characterization of safety, feasibility, and efficacy within cell culture and animal models, including a series of GLP pharmacology and toxicology studies using humanized mice and a non-human primate model needed for regulatory review. LVsh5/C46 is capable of mediating the expression of C46 and knockdown of CCR5 within target cells (Figure 2).

LVsh5/C46 lentiviral vector and LVsh5/C46 transduced CD34^+^ HSPC and CD4^+^ T-cells have strong safety profiles supported by *in vitro* and *in vivo* analysis including integration site analysis, inability to generate replication competent lentivirus (RCL), genomic stability, and maintenance of hematopoietic engraftment and multi-lineage differentiation of gene modified HSPC. Through all *in vitro*, *ex vivo* and *in vivo* studies, LVsh5/C46 has displayed a profound ability to confer cellular resistance to HIV infection.

## 8. Clinical Trial Design

**Figure 3:**
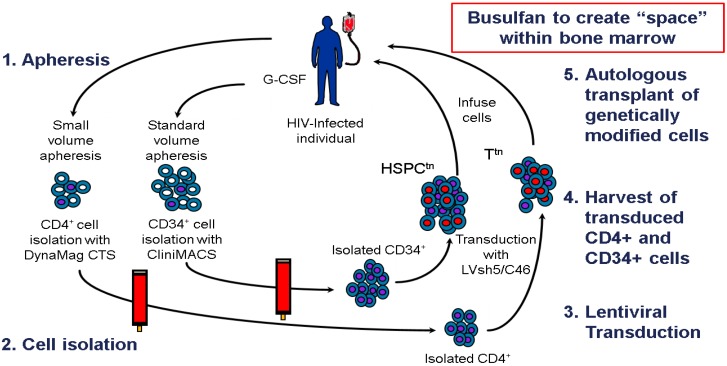
Schematic of the process for engineering protection from HIV-1 into human recipients via LVsh5/C46 mediated modification of CD4^+^ T lymphocytes and CD34^+^ HSPC. **1**. Apheresis, small or standard volume respectively, to obtain CD4^+^ T cells or CD34^+^ hematopoietic stem cells; **2**. Cell isolation using CliniMACS and DynaMag CTS bead separation; **3**. Lentiviral vector transduction with LVsh5/C46 in appropriate cytokines—CD3/CD28 bead stimulation and IL2 for T cells, stem cell factor, thrombopoietin and FLT-3 ligand for hematopoietic stem cells; **4**. Harvest of transduced cells and freezing; **5**. Following release testing, transplantation of genetically modified cells. Busulfan (4 mg/kg or 8 mg/kg) is administered pre cell infusion to make bone marrow space for introduced CD34^+^ HSPC.

Calimmune is conducting a Phase I/II clinical trial (CAL-USA-11) using LVsh5/C46 administered *ex vivo* using autologous CD4^+^ T lymphocytes and CD34^+^ HSPC in HIV-1 infected patients without malignancy. These cells are collected by separate apheresis procedures; one to collect CD4^+^ T lymphocytes and the other to collect CD34^+^ HSPC after mobilization with G-CSF. The therapeutic DNA is then integrated into the chromosomal DNA of a percentage of the collected cells rendering them and their progeny competent to express sh5 and C46. The LVsh5/C46 transduced CD4^+^ T lymphocytes (T^tn^) and LVsh5/C46 transduced CD34^+^ HSPC (HSPC^tn^) are then transplanted back to the patient where they have the potential to control HIV infection and stop disease progression (Figure 3).

Because the progression of HIV/AIDS depletes CD4^+^ T cells, a target of gene therapy approaches to date has been mature T lymphocytes. T lymphocytes are relatively abundant and are easily collected from peripheral blood, they can be readily transduced and expanded *ex vivo*; they also have the potential to rapidly replenish the immune system with HIV-protected cells and provide an immediate effect on viral load and T cell counts. Since CD4^+^ memory and effector T lymphocytes can persist for several years and have the capability for self-renewal [35,36], these cells also have the potential to provide longer-term therapeutic benefit. Early clinical data in the field of gene therapy has shown that delivery of genetically modified CD4^+^ T lymphocytes is safe and that the modified cells can have a survival advantage and sustained engraftment out to 10 years in some instances [33,36,37,38,39,40,41,42,43,44,45,46,47,48,49,50]. This strategy, however, cannot protect other susceptible hematopoietic cell types, such as macrophages and is less likely to affect latent HIV reservoirs.

An alternative approach has been the genetic modification of HSPC [51,52,53,54,55,56]. The advantage of using HSPC is their ability to self-renew and to differentiate into all hematopoietic cell types, endowing their progeny, CD4^+^ T lymphocytes, monocytes, macrophages, dendritic cells and microglia, the same genetic protection from HIV-1.

The combination of T^tn^ and HSPC^tn^, as being utilized by Calimmune, potentially provides the immediate benefit of large numbers of T^tn^ that will protect against HIV-1 during the immediate post-transplant engraftment and hematopoietic regeneration/differentiation period, with the long-term ability of the HSPC^tn^ to differentiate into all hematopoietic lineages and endow their progeny with the same genetic protection from HIV-1.

Clinical experience to date in the field of gene therapy suggests the potential for clinical benefit from gene-modified HSPC can be facilitated by creating an environment that enhances the ability of these cells to engraft and differentiate [57]. The potential of induction protocols, consisting of a non-myeloablative pre-transplant conditioning agent, will also be evaluated in the Calimmune trial for recipients of LVsh5/C46 transduced HSPC (Figure 3). As such the goal of the trial will not only be to evaluate the safety and feasibility of using LVsh5/C46 transduced CD34^+^ HSPC and CD4^+^ T lymphocytes, but will also look at the use of busulfan as a non-myeloablative conditioning agent to increase LVsh5/C46 transduced CD34^+^ HSPC engraftment. Secondarily, the trial will assess the level of gene marked cells and any impact on HIV-1 RNA and CD4^+^ T lymphocyte counts, as well as other exploratory parameters.

## 9. Conclusions: Tying It All Together

### 9.1. CCR5-Delta32 Compared with Down-Regulation of CCR5

The “Berlin patient” showed that modulating CCR5 can lead to a functional cure of HIV. In that case CCR5-delta32 hematopoietic stem cells were introduced following myeloablative conditioning leading to T cell recovery and suppression of HIV. This approach required a matched donor that was CCR5-delta32. This review documents the difficulty in repeating the approach of transplanting allogeneic CCR5-delta32 donor cells. Calimmune is testing the use of a short hairpin RNA to CCR5 to reproduce this result using autologous cells that are genetically modified *ex vivo*. In this approach, CCR5 is down regulated by the action of the shRNA to CCR5 and an additional agent, C46, is employed to increase efficacy and to mitigate the development of resistance.

### 9.2. Durability of Response

The “Berlin patient” exhibited a durable response in that T cells were reconstituted and HIV suppressed in an ongoing manner. The approach of Calimmune is being tested in a Phase I/II clinical trial to determine whether the introduction of a dual therapeutic can lead to significant numbers of autologous gene modified cells that are protected from HIV infection and pathogenicity.
